# Chloral and Cod Liver Oil

**Published:** 1871-12

**Authors:** 


CHLORAL IN COD-LIVER OIL is said to render it much less nauseous, and prevents the night-sweats of the phthisical patient induces sleep, and creates appetite. The pure chloral-hydrate crystals may be added to cod-liver oil in the proportion of ten grains of the former to one hundred and ninety of the latter.—*Medical Times.*

